# Supplementary material for: Predicting Flow Reversals in a Computational Fluid Dynamics Simulated Thermosyphon Using Data Assimilation
Source: PLoS One. 2016 Feb 5;11(2):e0148134. doi: 10.1371/journal.pone.0148134 (PMC4743928; doi:10.1371/journal.pone.0148134)
Supplement: S1 Appendix — (PDF) [file pone.0148134.s001.pdf]

Supporting Information

S1 Appendix: Computational Details and Explicit Equations Used

In this section, we first present the governing equations for the flow in our thermal convection loop experiment. A spatial and temporal discretization of the governing equations is then necessary so that they may be solved numerically. After discretization, we must specify the boundary conditions. With the mesh and boundary conditions in place, we can then simulate the flow with a computational fluid dynamics solver.

We now discuss the equations, mesh, boundary conditions, and solver in more detail. With these considerations, we present our simulations of the thermosyphon. For a complete derivation of the equations used, see [35].

We consider the incompressible Navier-Stokes equations with the Boussinesq approximation to model the flow of water inside a thermal convection loop. Here we present the main equations that are solved numerically, noting the assumptions that are necessary in their derivation. In standard notation, for u, v, w the velocity in the x, y, z direction, respectively, the continuity equation for an incompressible fluid is

$$\frac{\partial u}{\partial x} + \frac{\partial v}{\partial y} + \frac{\partial w}{\partial z} = 0. \tag{S1}$$

The momentum equations, in tensor notation with bars representing averaged quantities (long timesteps are used, requiring integration), are

$$\rho_{\text{ref}} \left(\frac{\partial \bar{u}_i}{\partial t} + \frac{\partial}{\partial x_j} \left(\bar{u}_j \bar{u}_i \right) \right) = -\frac{\partial \bar{p}}{\partial x_i} + \mu \frac{\partial \bar{u}_i}{\partial x_j^2} + \rho_{\text{ref}} \left(1 - \beta (T - T_{\text{ref}}) \right) g_i \tag{S2}$$

for ρ_{ref} the reference density with the Boussinesq approximation included, p the pressure, μ the viscosity, and g_i gravity in the i-direction. Note that $g_i = 0$ for $i \in \{x, y\}$ since gravity is assumed to be the z direction. Since the model is incompressible, of course our energy equation includes only temperature, and is given by

$$\frac{\partial T}{\partial t} + \frac{\partial}{\partial x_j} \left(\rho_{\text{ref}} T \overline{u}_j \right) - \frac{\partial^2 \alpha T}{\partial x_j \partial x_i} = -\frac{\partial q_k^*}{\partial x_k} - \frac{\partial \overline{q}_k}{\partial x_k} \tag{S3}$$

for T the temperature and q the flux (where $q = \overline{q} + q^*$ is the averaging notation).

The PISO (Pressure-Implicit with Splitting of Operators) algorithm derives from the work of [23], and is complementary to the SIMPLE (Semi-Implicit Method for Pressure-Linked Equations) [36] iterative method. The main difference of the PISO and SIMPLE algorithms is that in the PISO, no under-relaxation is applied and the momentum corrector step is performed more than once [37]. They sum up the algorithm in nine steps:

- Set the boundary conditions.
- Solve the discretized momentum equation to compute an intermediate velocity field.
- Compute the mass fluxes at the cell faces.
- Solve the pressure equation.
- Correct the mass fluxes at the cell faces.
- Correct the velocity with respect to the new pressure field.

- Update the boundary conditions.
- Repeat from step #3 for the prescribed number of times.
- Repeat (with increased time step).

The solver itself has 647 dependencies, of which I present only a fraction. The main code is straight forward, relying on include statements to load the libraries and equations to be solved.

```
#include "fvCFD.H"
#include "singlePhaseTransportModel.H"
#include "RASModel.H" // AJR edited header 2013-10-14
#include "radiationModel.H" // (model loaded but not used)
#include "fvIOoptionList.H" // (loaded, but also not used)
#include "pimpleControl.H"
```

The main function is then

```
int main(int argc, char *argv[])
{
    #include "setRootCase.H"
    #include "createTime.H"
    #include "createMesh.H"
    #include "readGravitationalAcceleration.H"
    #include "createFields.H"
    #include "createIncompressibleRadiationModel.H"
    #include "createFvOptions.H"
    #include "initContinuityErrs.H"
    #include "readTimeControls.H"
    #include "CourantNo.H"
    #include "setInitialDeltaT.H"
    pimpleControl pimple(mesh);
```

We then enter the main loop. This is computed for each time step, prescribed before the solver is applied. Note that the capacity is available for adaptive time steps, choosing to keep the Courant number below some threshold, but I do not use this. For the distributed ensemble of model runs, it is important that each model complete in nearly the same time, so that the analysis is not waiting on one model and therefore under-utilizing the available resources.

```
    while (runTime.loop())
    {
        #include "readTimeControls.H"
        #include "CourantNo.H"
        #include "setDeltaT.H"
        while (pimple.loop())
        {
            #include "UEqn.H"
            #include "TEqn.H"
            while (pimple.correct())
            {
                #include "pEqn.H"
            }
        }
        if (pimple.turbCorr())
        {
            turbulence->correct();
        }
```

Opening up the equation for U we see that Equation

```
    // Solve the momentum equation
    fvVectorMatrix UEqn
    (
        fvm::ddt(U)
      + fvm::div(phi, U)
      + turbulence->divDevReff(U)
     ==
        fvOptions(U)
```

```
    );
    UEqn.relax();
    fvOptions.constrain(UEqn);
    if (pimple.momentumPredictor())
    {
        solve
        (
            UEqn
         ==
            fvc::reconstruct
            (
                (
                  - ghf*fvc::snGrad(rhok)
                  - fvc::snGrad(p_rgh)
                )*mesh.magSf()
            )
        );
        fvOptions.correct(U);
    }
```

Solving for T is

```
{
    // with our laminar model, alphat = 0
    alphat = turbulence->nut()/Prt;
    alphat.correctBoundaryConditions();
    volScalarField alphaEff("alphaEff", turbulence->nu()/Pr + alphat);
    fvScalarMatrix TEqn
    (
        fvm::ddt(T)
      + fvm::div(phi, T)
      - fvm::laplacian(alphaEff, T)
     ==
        radiation->ST(rhoCpRef, T)
      + fvOptions(T)
    );
    TEqn.relax();
    fvOptions.constrain(TEqn);
    TEqn.solve();
    radiation->correct();
    fvOptions.correct(T);
    rhok = 1.0 - beta*(T - TRef); // Boussinesq approximation
}
```

Finally, we solve for the pressure p in “pEqn.H”:

```
{
    volScalarField rAU("rAU", 1.0/UEqn.A());
    surfaceScalarField rAUf("Dp", fvc::interpolate(rAU));
    volVectorField HbyA("HbyA", U);
    HbyA = rAU*UEqn.H();
    surfaceScalarField phig(-rAUf*ghf*fvc::snGrad(rhok)*mesh.magSf());
    surfaceScalarField phiHbyA
    (
        "phiHbyA",
        (fvc::interpolate(HbyA) & mesh.Sf())
      + fvc::ddtPhiCorr(rAU, U, phi)
      + phig
    );
    while (pimple.correctNonOrthogonal())
    {
        fvScalarMatrix p_rghEqn
        (
            fvm::laplacian(rAUf, p_rgh) == fvc::div(phiHbyA)
        );
        p_rghEqn.setReference(pRefCell, getRefCellValue(p_rgh, pRefCell));
        p_rghEqn.solve(mesh.solver(p_rgh.select(pimple.finalInnerIter())));
        if (pimple.finalNonOrthogonalIter())
        {
            // Calculate the conservative fluxes
            phi = phiHbyA - p_rghEqn.flux();
            // Explicitly relax pressure for momentum corrector
            p_rgh.relax();
            // Correct the momentum source with the pressure gradient flux
            // calculated from the relaxed pressure
            U = HbyA + rAU*fvc::reconstruct((phig - p_rghEqn.flux())/rAUf);
            U.correctBoundaryConditions();
```

```
        }
    }
    #include "continuityErrs.H"
    p = p_rgh + rhok*gh;
    if (p_rgh.needReference())
    {
        p += dimensionedScalar
        (
            "p",
            p.dimensions(),
            pRefValue - getRefCellValue(p, pRefCell)
        );
        p_rgh = p - rhok*gh;
    }
}
```

The final operation being the conversion of pressure to hydrostatic pressure,

$$p_{\mathrm{rgh}} = p - \rho_k g_h.$$

This "pEqn.H" is then re-run until convergence is achieved, and the PISO loop begins again.

We verify convergence of the solution in a steady flow state regime in Fig S1.

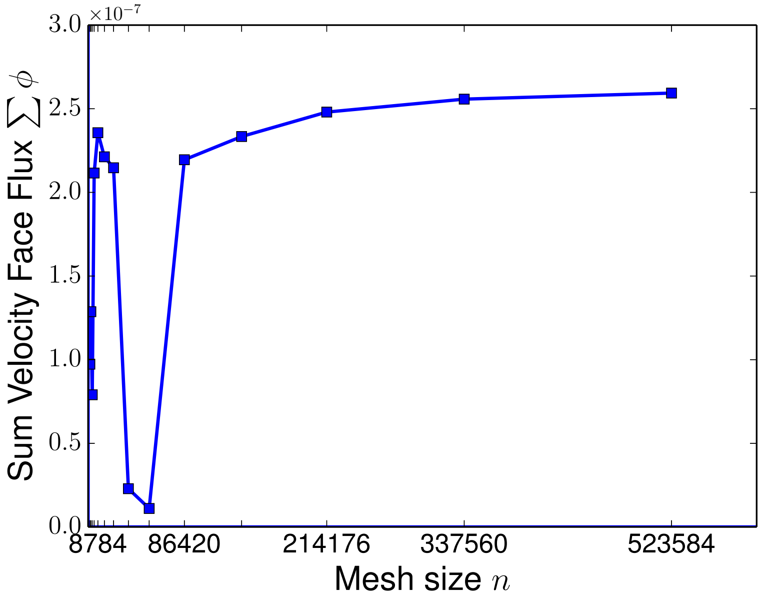

Figure S1. With a fixed choice of solver, boundary conditions, and initial conditions that lead to a stable convective state, we present the long-term behavior of the velocity face flux at the top slice for different meshes. The face flux is reported as the average for the last 20 times saves for which the velocity flux is summed across a slice perpendicular to the loop, here we show the top slice. We choose a fixed time step of 0.005 for each simulation, and run the solver for 60 hours on 8 cores. The computational limit of mesh creation was a memory limit at 818280 cells, so we present results for meshes starting at 1600 cells and cells decreasing in size by a factor of 1.25 in both y and z up to a mesh of 523584 cells. For meshes with more than 80,000 cells we see that the solutions are very similar. The smaller meshes generate increasing unstable flow behavior, leading to oscillations of flux and then flow reversals for the smallest meshes of size 2500 and 1600 cells.
