# Supplementary material for: Predicting Flow Reversals in a Computational Fluid Dynamics Simulated Thermosyphon Using Data Assimilation
Source: PLoS One. 2016 Feb 5;11(2):e0148134. doi: 10.1371/journal.pone.0148134 (PMC4743928; doi:10.1371/journal.pone.0148134)
Supplement: S2 Appendix — (PDF) [file pone.0148134.s002.pdf]

S2 Appendix: The Ehrhard and Müller Equations

Following the derivation by Harris [8], itself a representation of the derivation of Gorman [13] and namesakes Ehrhard and Müller [14], we derive the equations governing a closed loop thermosyphon.

Similar to the derivation of the governing equations of computational fluid dynamics, we start with a small but finite volume inside the loop. Here, however, the volume is described by $\pi r^2 R \mathrm{d}\phi$ for r the interior loop size (such that πr^2 is the area of a slice) and $R\mathrm{d}\phi$ the arc length (width) of the slice. Newton's second law states that momentum is conserved, such that the sum of the forces acting upon our finite volume is equal to the change in momentum of this volume. Therefore we have the basic starting point for forces $\sum F$ and velocity u as

$$\sum F = \rho \pi r^2 R \mathrm{d}\phi \frac{\mathrm{d}u}{\mathrm{d}t}. \tag{S4}$$

The sum of the forces is $\sum F = F_{\{p,f,g\}}$ for net pressure, fluid shear, and gravity, respectively. We write these as

$$F_p = -\pi r^2 \mathrm{d}\phi \frac{\partial p}{\partial \phi} \tag{S5}$$

$$F_w = -\rho \pi r^2 \mathrm{d}\phi f_w \tag{S6}$$

$$F_g = -\rho \pi r^2 \mathrm{d}\phi g \sin(\phi) \tag{S7}$$

where $\partial p/\partial\phi$ is the pressure gradient, f_w is the wall friction force, and $g\sin(\phi)$ is the vertical component of gravity acting on the volume.

We now introduce the Boussinesq approximation which states that both variations in fluid density are linear in temperature T and density variation is insignificant except when multiplied by gravity. The consideration manifests as

$$\rho = \rho(T) \simeq \rho_{\mathrm{ref}}(1 - \beta(T - T_{\mathrm{ref}})$$

where ρ_0 is the reference density and T_{ref} is the reference temperature, and β is the thermal expansion coefficient. The second consideration of the Boussinesq approximation allows us to replace ρ with this ρ_{ref} in all terms except for F_g. We now write momentum equation as

$$-\pi r^2 \mathrm{d}\phi \frac{\partial p}{\partial \phi} - \rho_{\mathrm{ref}} \phi r^2 R \mathrm{d}\phi f_w - \rho_{\mathrm{ref}}(1 - \rho(T - T_{\mathrm{ref}}))\pi r^2 R \mathrm{d}\phi g \sin(\phi) = \rho_{\mathrm{ref}} \pi r^2 R \mathrm{d}\phi \frac{\mathrm{d}u}{\mathrm{d}t}. \tag{S8}$$

Canceling the common πr^2, dividing by R, and pulling out $\mathrm{d}\phi$ on the LHS we have

$$-\mathrm{d}\phi \left(\frac{\partial p}{\partial \phi} \frac{1}{R} - \rho_{\mathrm{ref}} f_w - \rho_{\mathrm{ref}}(1 - \rho(T - T_{\mathrm{ref}}))g \sin(\phi) \right) = \rho_{\mathrm{ref}} \mathrm{d}\phi \frac{\mathrm{d}u}{\mathrm{d}t}. \tag{S9}$$

We integrate this equation over ϕ to eliminate many of the terms, specifically we have

$$\int_0^{2\pi} -\mathrm{d}\phi \frac{\partial p}{\partial \phi} \frac{1}{R} \to 0$$

$$\int_0^{2\pi} -\mathrm{d}\phi \rho_{\mathrm{ref}} g \sin(\phi) \to 0$$

$$\int_0^{2\pi} -\mathrm{d}\phi \rho_{\mathrm{ref}} \beta T_{\mathrm{ref}} g \sin(\phi) \to 0.$$

Since u (and hence $\frac{\mathrm{d}u}{\mathrm{d}\phi}$) and f_w do not depend on ϕ, we can pull these outside an integral over ϕ and therefore the momentum equation is now

$$2\pi f_w \rho_0 + \int_0^{2\pi} \mathrm{d}\phi \rho_{\mathrm{ref}} \beta T g \sin(\phi) = 2\pi \frac{\mathrm{d}u}{\mathrm{d}\phi} \rho_{\mathrm{ref}}.$$

Diving out 2π and pull constants out of the integral we have our final form of the momentum equation

$$f_w \rho_{\mathrm{ref}} + \frac{\rho_{\mathrm{ref}} \beta g}{2\pi} \int_0^{2\pi} \mathrm{d}\phi T \sin(\phi) = \frac{\mathrm{d}u}{\mathrm{d}\phi} \rho_{\mathrm{ref}}. \tag{S10}$$

Now considering the conservation of energy within the thermosyphon, the energy change within a finite volume must be balanced by transfer within the thermosyphon and to the walls. The internal energy change is given by

$$\rho_{\mathrm{ref}} \pi r^2 R \mathrm{d}\phi \left(\frac{\partial T}{\partial t} + \frac{u}{R} \frac{\partial T}{\partial \phi} \right) \tag{S11}$$

which must equal the energy transfer through the wall, which is, for T_w the wall temperature:

$$\dot{q} = -\pi r^2 R \mathrm{d}\phi h_w (T - T_w). \tag{S12}$$

Combining Equations S11 and S12 (and canceling terms) we have the energy equation:

$$\left(\frac{\partial T}{\partial t} + \frac{u}{R} \frac{\partial T}{\partial \phi} \right) = \frac{-h_w}{\rho_{\mathrm{ref}} c_p} \left(T - T_w \right). \tag{S13}$$

The f_w which we have yet to define and h_w are fluid-wall coefficients and can be described by [14]:

$$h_w = h_{w_0} \left(1 + K h(|x_1|) \right)$$
$$f_w = \frac{1}{2} \rho_{\mathrm{ref}} f_{w_0} u.$$

We have introduced an additional function h to describe the behavior of the dimensionless velocity $x_1 \alpha u$. This function is defined piece-wise as

$$h(x) = \begin{cases} x^{1/3} & \text{when } x \geq 1 \\ p(x) & \text{when } x < 1 \end{cases} \tag{S14}$$

where $p(x)$ can be defined as $p(x) = \left(44x^2 - 55x^3 + 20x^4\right)/9$ such that p is analytic at 0 [8].

Taking the lowest modes of a Fourier expansion for T for an approximate solution, we consider:

$$T(\phi, t) = C_0(t) + S(t) \sin(\phi) + C(t) \cos(\phi). \tag{S15}$$

By substituting this form into Equations S10 and S13 and integrating, we obtain a system of three equations for our solution. We then follow the particular nondimensionalization choice of Harris *et al.*such that we obtain the following ODE system, which we refer to as the Ehrhard-Müller equations:

$$\frac{\mathrm{d}x_1}{\mathrm{d}t'} = \alpha(x_2 - x_1), \tag{S16}$$

$$\frac{\mathrm{d}x_2}{\mathrm{d}t'} = \beta x_1 - x_2(1 + K h(|x_1|)) - x_1 x_3, \tag{S17}$$

$$\frac{\mathrm{d}x_3}{\mathrm{d}t'} = x_1 x_2 - x_3(1 + K h(|x_1|)). \tag{S18}$$

The nondimensionalization is given by the change of variables

$$t' = \frac{h_{w_0}}{\rho_{\mathrm{ref}} c_p} t, \tag{S19}$$

$$x_1 = \frac{\rho_{\mathrm{ref}} c_p}{R h_{w_0}} u, \tag{S20}$$

$$x_2 = \frac{1}{2} \frac{\rho_{\mathrm{ref}} c_p \beta g}{R h_{w_0} f_{w_0}} \Delta T_{3-9}, \tag{S21}$$

$$x_3 = \frac{1}{2} \frac{\rho_{\mathrm{ref}} c_p \beta g}{R h_{w_0} f_{w_0}} \left(\frac{4}{\pi} \Delta T_w - \Delta T_{6-12} \right) \tag{S22}$$

and

$$\alpha = \frac{1}{2} R c_p f_{w_0} / h_{w_0}, \tag{S23}$$

$$\gamma = \frac{2}{\pi} \frac{\rho_{\mathrm{ref}} c_p \beta g}{R h_{w_0} f_{w_0}} \Delta T_w. \tag{S24}$$

Through careful consideration of these non-dimensional variable transformations we verify that x_1 is representative of the mean fluid velocity, x_2 of the temperature difference between the 3 and 9 o'clock positions on the thermosyphon, and x_3 the deviation from the vertical temperature profile in a conduction state [8].
