# Supplementary material for: Predicting Flow Reversals in a Computational Fluid Dynamics Simulated Thermosyphon Using Data Assimilation
Source: PLoS One. 2016 Feb 5;11(2):e0148134. doi: 10.1371/journal.pone.0148134 (PMC4743928; doi:10.1371/journal.pone.0148134)
Supplement: S3 Appendix — (PDF) [file pone.0148134.s003.pdf]

S3 Appendix: Data Assimiliation

The TLM is the model which advances an initial perturbation $\delta\mathbf{x}_i$ at timestep i to a final perturbation $\delta\mathbf{x}_{i+1}$ at timestep $i+1$. The dynamical system we are interested in, Lorenz '63, is given as a system of ODE's:

$$\frac{d\mathbf{x}}{dt} = F(\mathbf{x}).$$

We integrate this system using a numerical scheme of our choice (in the given examples we use a second-order Runge-Kutta method), to obtain a model M discretized in time.

$$\mathbf{x}(t) = M[\mathbf{x}(t_0)].$$

Introducing a small perturbation $\mathbf{y}$, we can approximate our model M applied to $\mathbf{x}(t_0) + \mathbf{y}(t_0)$ with a Taylor series around $\mathbf{x}(t_0)$:

$$\begin{aligned} M[\mathbf{x}(t_0) + \mathbf{y}(t_0)] &= M[\mathbf{x}(t_0)] + \frac{\partial M}{\partial \mathbf{x}}\mathbf{y}(t_0) + O[\mathbf{y}(t_0)^2] \\ &\approx \mathbf{x}(t) + \frac{\partial M}{\partial \mathbf{x}}\mathbf{y}(t_0). \end{aligned}$$

We can then solve for the linear evolution of the small perturbation $\mathbf{y}(t_0)$ as

$$\frac{d\mathbf{y}}{dt} = \mathbf{J}\mathbf{y} \tag{S25}$$

where $\mathbf{J} = \partial F/\partial \mathbf{x}$ is the Jacobian of F. We can solve the above system of linear ordinary differential equations using the same numerical scheme as we did for the nonlinear model.

One problem with solving the system of equations given by Equation S25 is that the Jacobian matrix of discretized code is not necessarily identical to the discretization of the Jacobian operator for the analytic system. This is a problem because we need to have the TLM of our model M, which is the time-space discretization of the solution to $d\mathbf{x}/dt = F(\mathbf{x})$. We can apply our numerical method to the $d\mathbf{x}/dt = F(\mathbf{x})$ to obtain M explicitly, and then take the Jacobian of the result. This method is, however, prohibitively costly, since Runge-Kutta methods are implicit. It is therefore desirable to take the derivative of the numerical scheme directly, and apply this differentiated numerical scheme to the system of equations $F(\mathbf{x})$ to obtain the TLM. A schematic of this scenario is illustrated in Figure S2. To that the derivative of numerical code for implementing the EKF on models larger than 3 dimensions (i.e. global weather models written in Fortan), automatic code differentiation is used [38].

To verify our implementation of the TLM, we propagate a small error in the Lorenz '63 system and plot the difference between that error and the TLM predicted error, for each variable (Figure S3).

With a finite ensemble size, the ensemble method is only an approximation and therefore in practice it often fails to capture the full spread of error. To better capture the model variance, additive and multiplicative inflation factors are used to obtain a good estimate of the error covariance matrix (Data Assimilation Section). The spread of ensemble members in the x_1 variable of the Lorenz model, as distance from the analysis, can be seen in Figure S4.

In computing the error covariance $\mathbf{P}_f$ from the ensemble, we wish to add up the error covariance of each forecast with respect to the mean forecast. But this would underestimate the error covariance, since the forecast we're comparing against is used in the ensemble average (to obtain the mean forecast). Therefore, to compute the error

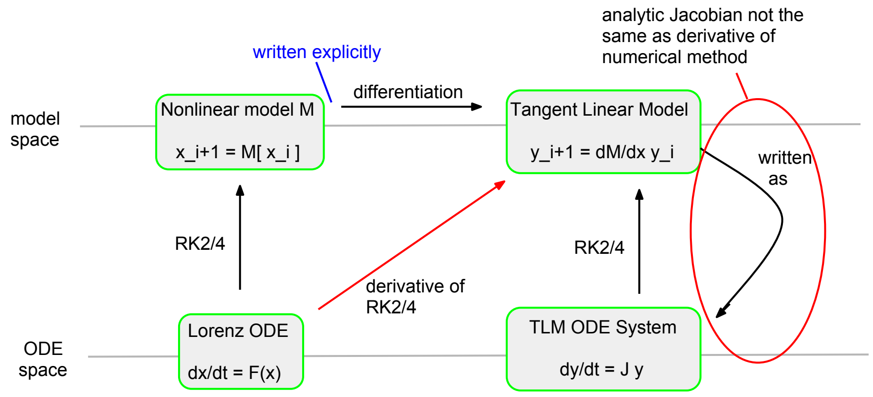

Figure S2. An explanation of how and why the best way to obtain a TLM is with a differentiated numerical scheme. Both the Lorenz ODE and TLM ODE System can be solved by RK2/4, but the analytic Jacobian of TLM that would is not the same as the derivative of the numerical method. In particular, the derivative of the RK2/4 integrator is used to obtain a TLM that most accurately propogates error growth in the Lorenz '63 system.

covariance matrix for each forecast, that forecast itself is excluded from the ensemble average forecast.

We can see the classic spaghetti of the ensemble with this filter implemented on Lorenz 63 in Figure S5.

We denote the forecast within an ensemble filter as the average of the individual ensemble forecasts, and an explanation for this choice is substantiated by Burgers [39]. The general EnKF which we use is most similar to that of Burgers. Many algorithms based on the EnKF have been proposed and include the Ensemble Transform Kalman Filter (ETKF) [31], Ensemble Analysis Filter (EAF) [40], Ensemble Square Root Filter (EnSRF) [41], Local Ensemble Kalman Filter (LEKF) [31], and the Local Ensemble Transform Kalman Filter (LETKF) [32]. A comprehensive overview through 2003 is provided by Evensen [42]. For further details on the most advanced methods, beyond what is provided in the body of the paper, we direct the reader the above references and the derivations provided in [35].

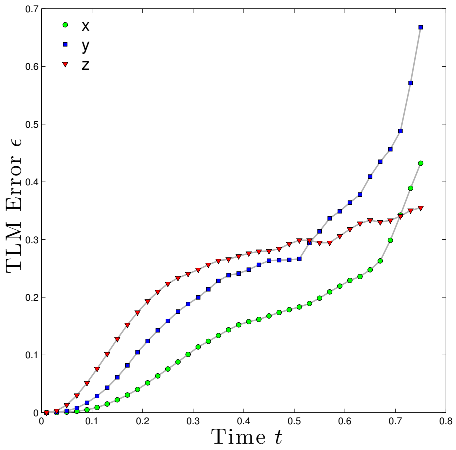

Figure S3. The future error predicted by the TLM is compared to the error growth in Lorenz '63 system for an initial perturbation with standard deviation of 0.1, averaged over 1000 TLM integrations. The ϵ is not the error predicted by the TLM, but rather the error of the TLM in predicting the error growth. We see an intially linear error growth for small time, which is overcome by the nonlinearity of the Lorenz system for longer time.

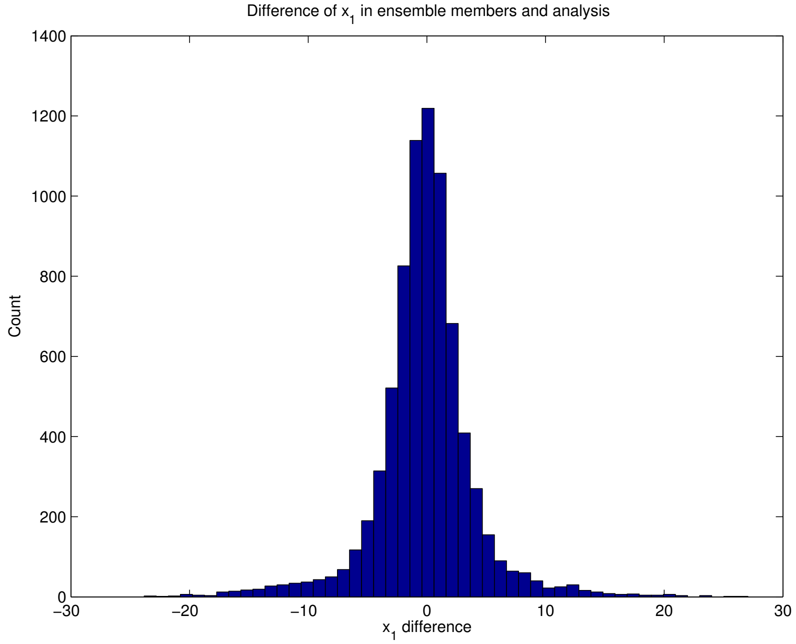

Figure S4. The difference of ensemble forecasts from the analysis is reported for 760 assimilation windows in one model run of length 200, with 10 ensemble members and an assimilation window of length 0.261. This has the same shape of as the difference between ensemble forecasts and the mean of the forecasts (not shown). This spread of ensemble forecasts is what allows us to estimate the error covariance of the forecast model, and appears to be normally distributed.

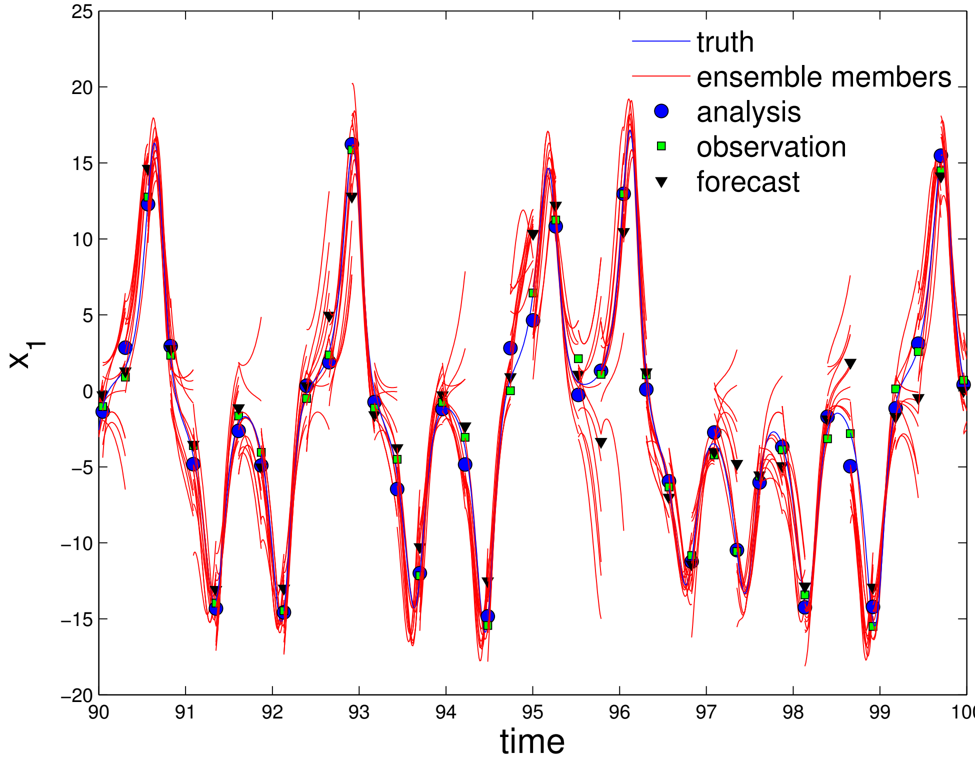

Figure S5. A sample time-series of the ensembles used in the EnKF. In all tests, as seen here, 10 ensemble members are used. For this run, 384 assimilation cycles are performed with a window length of 0.26 model time units. We can see that the ensemble member state after assimilation better represents the uncertainty of the analysis state and enables some ensemble members to stay close to the true state.
