# Supplementary material for: Predicting Flow Reversals in a Computational Fluid Dynamics Simulated Thermosyphon Using Data Assimilation
Source: PLoS One. 2016 Feb 5;11(2):e0148134. doi: 10.1371/journal.pone.0148134 (PMC4743928; doi:10.1371/journal.pone.0148134)
Supplement: S4 Appendix — (PDF) [file pone.0148134.s004.pdf]

S4 Appendix: Additional DMD Details

The general algorithm for DMD is provided in the Dynamic Mode Decomposition Section, and here we supply more results of the DMD procedure. The timeseries from which we computed the decomposition is shown in Figure S6.

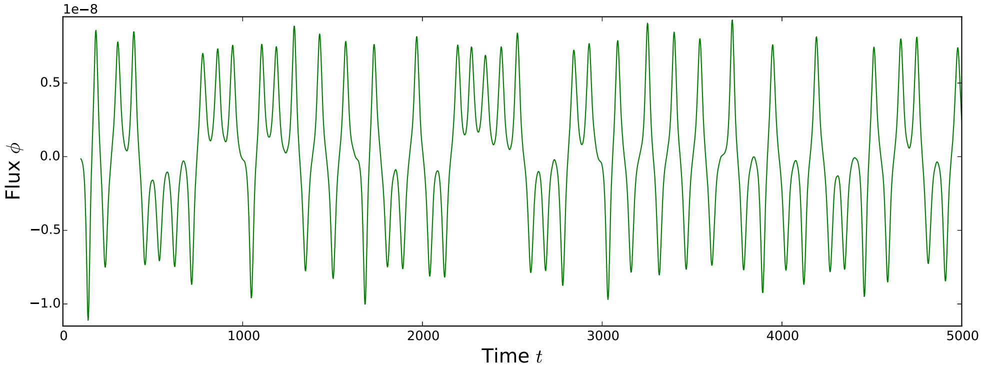

Figure S6. Flux timeseries on which DMD is performed. We report the flux as the sum of the face flux values on a slice of the loop at the 9 o'clock position. In this flux timeseries we see dynamics visually similar to the x_1 variable of the Lorenz 63 system. Residence time in either flow direction is aperiodic and unstable with the flow speed oscillating within a single direction with growing amplitude until reversing.

The real and imaginary components of the DMD eigenvalues are shown in both un-mapped and mapped form in Figure S7.

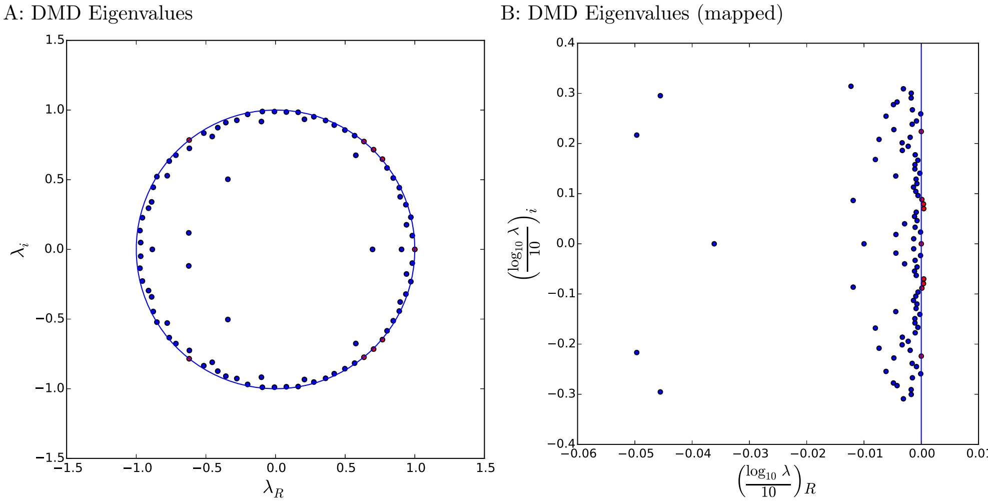

Figure S7. Eigenvalues of DMD Modes presented in both raw and scaled (mapped) form. In Panel A we see the eigenvalue of each DMD mode on the complex plane, with an inset unit circle. Those eigenvalues with magnitude greater than 1 are shown in red. In Panel B we see the same eigenvalues on the complex plane, transformed by the base 10 logarithm. Again we color in red those eigenvalues with real part greater than 0.
